# Development and perceived usability evaluation of an interactive smartphone application for the teaching of hemodynamics and evaluation of arterial pulse pressure variation

**DOI:** 10.1186/s12911-023-02131-5

**Published:** 2023-02-09

**Authors:** Orivaldo Alves Barbosa, Edgar Marçal, David Augusto Batista Sá Araújo, Lucas Severo Melo, Hermano Alexandre Lima Rocha

**Affiliations:** 1 Unichristus University Center, Rua Papi Junior, 1223, 5th Floor, Fortaleza, CE 0430-235 Brazil; 2 Federal University of Ceará, Fortaleza, CE Brazil; 3 Department of Global Health and Population, Harvard T. H. Chan School of Public Health, Boston, MA USA

**Keywords:** Critical care, Mobile applications, Hemodynamic monitoring, Fluid responsiveness

## Abstract

**Background:**

The use of fluids is the most frequently used therapy for circulatory shock. Its inadequate use has adverse effects, requiring hemodynamic goals, highlighting the use of pulse pressure variation due to its high accuracy. One of the problems related to this method is the difficulties in measuring the pulse pressure variation (PPV) in most monitors for clinical use. We assessed the qualitative aspects of perceived usability of a smartphone application (app), which, based on a photograph of the patient's arterial pulse wave, can help measure PPV and help in the diagnosis and management of shock cases.

**Methods:**

To assess the software perceived usability, we used the System Usability Scale (SUS) applied to 30 physicians in 2 tertiary hospitals in Brazil. The software accuracy was measured using a sequence of 3 images with different values ​​of pulse pressure variation, comparing the obtained values ​​ with the gold standard. The educational interface of the app was evaluated qualitativelyfrom the spontaneous testimonies of the selected test participants.The project was approved by the Research Ethics Committee of Centro Universitário Christus.

**Results:**

The analysis showed an average SUS of 86.3 points on a scale of 1–100 (above 80.3 is considered the best in terms of interface). The assessment of the application's accuracy when evaluating pulse pressure variation showed that the average variation of the measurements taken by the participants was small, with a good measure of repeatability and reproducibility. The app's educational interface was qualitatively evaluated, being praised by the users.

**Conclusions:**

It can be concluded that the developed mobile application showed excellent qualitative aspects of perceived usability results. More studies with this app will be required to evaluate the potential to help professionals with hemodynamic evaluation in emergency and intensive care settings.

## Background

Shock is a common condition in critical care, affecting approximately a third of the patients in the intensive care unit (ICU) [1]. Early and adequate hemodynamic support of patients in shock is crucial to prevent organ dysfunction deterioration and failure. Fluid therapy aiming to improve microvascular blood flow and increase cardiac output is an essential part of the treatment of any type of shock, but despite being the most widely used treatment in intensive care worldwide, fluid resuscitation is a frequent source of doubts, diagnostic and therapeutic errors and academic questions in the ICU [2–4].

Plasma expansion with intravenous fluids is the most frequently used treatment in most cases of shock; however, excess fluid leads to increased mortality in critically-ill patients, especially septic patients, due to increased mechanical ventilation time, acute kidney injury, respiratory distress syndrome and intra-abdominal hypertension. On the other hand, in cases of fluid responsiveness in hypotensive patients, its use improves cardiac output and systemic perfusion, with an impact on mortality [5–7].

Among the several hemodynamic parameters that have been described, pulse pressure variation (PPV), derived from the analysis of the arterial waveform and its contour, has shown to be the best predictor of fluid responsiveness when performed under ideal conditions (patients in sinus rhythm, intubated and mechanically ventilated, not making any spontaneous respiratory efforts, with at least 8 mL/kg of tidal volume, without changes in chest wall compliance) [8]. A recent meta-analysis that included 22 studies showed a sensitivity of 88% with a specificity of 89% [9]. The formula, initially described by Michard et al. is as follows:

$$\cdot \;{\text{PPV}} = {1}00 \times \left( {{\text{PPmax}}{-}{\text{Ppmin}}} \right)/{\text{PPmax}} + {\text{PPmin}}/{2}$$

where PPmax is the highest pulse pressure in the inspiratory period and PPmin is the lowest pulse pressure in the expiratory period.

PPV is a reflection of cardiopulmonary interactions. Cardiac output varies when a patient breathes both spontaneously and on mechanical ventilation. The more the cardiac output varies with breaths, the more likely the patient is to respond to fluids with an increase in stroke volume. Using this simple principle, clinicians can use the common arterial line tracing to assess the patient's responsiveness to volume [10]. Despite being technically easy to perform and cost-effective, PPV is underutilized in clinical practice due to the lack of knowledge of the variable by the physicians or, mainly, due to the difficulty in measuring it in conventional use clinical monitors. Some arterial catheters with specific transducers that analyze the pulse wave contour based on automated algorithms generate the stroke volume variation (SVV) continuously, but at a high cost, making its routine application difficult [11].

The cost associated with modern intensive care is extremely high and new modalities that assist monitoring and decision making at low cost are therefore essential, especially in low-income regions such as Brazil. With the expansion of mobile phone technology comes a new opportunity for potentially cost-effective monitoring systems for intensive care interventions. We assessed the qualitative aspects of perceived usability of an application for mobile devices aiming to assist in the optimization of fluid therapy in critically-ill patients, helping to measure the arterial pulse pressure variation, evaluating its usability and measurement accuracy.

## Methods

The app for mobile devices was developed for Android and iOS, using the Java language, with the participation of a multidisciplinary team consisting of two professors from the medical course and one from Computer Sciences, a systems analyst, a computer programmer and a graphic designer. Software development kits (SDKs) were used for specific Android and Apple devices. For Android platforms, the Android Studio's IDE (Integrated Development Environment) tools, Google's Android with APIs (Application Programming Interface) and the OpenCV (Open-Source Computer Vision) library were used. For the development, a work team was assembled with the authors and the programming team. A flow of activities was established, which involved monthly meetings from the creation of the prototype version on. At each meeting, demands were identified and passed on to the software programmer and incorporated into the app. Improvement suggestions and qualitative feedback made by users in the testing phases were incorporated into the program after the final analysis.

Satisfaction with perceived usability was assessed based on the version of the System Usability Scale (SUS) translated into Brazilian Portuguese [12]. The SUS contains ten questions that aim to measure the usability of different products and services, being technologically versatile to evaluate various products and services [13], including websites, hardware, multimodal systems, voice command systems, mobile applications and clinical systems [14]. The original version of SUS was developed by John Brooke in 1986 as part of a usability engineering program to allow the user to evaluate the usability of a particular product or service quickly and easily. When SUS is used, participants are asked to score the following 10 items with one of five responses that range from Strongly Agree to Strongly disagree (see Table 2 for the 10 questions contained in the SUS survey). To estimate usability, the contributions of each scoring item were added together. For the positively worded questions [1, 3, 5, 7, 9], the score contribution is the answer value minus 1. For the negatively worded questions [2, 4, 6, 8, 10], the contribution is equal to 5 minus the answer value. Subsequently, the values of the contributions of the 10 questions are added and multiplied by 2.5 to obtain the value of the SUS score.

Regarding the classification obtained by the score

-  < 20.5 (worst imaginable)
- 21 to 38.5 (poor)
- 39.5 to 52 (average)
- 53 to 73.5 (good)
- 74 to 85.5 (excellent)
- Greater than 86 (best imaginable)

After the evaluation by the SUS scale, participants were directly interviewed to provide an evaluation of positive and negative aspects, as well as suggestions for improvements in the use of the app, and they could freely answer this question.

After presenting the app installed on a smartphone, we made a brief explanation to each participant about the aim the research and the pulse pressure variation. To assess usability, the researcher initially operated the app and used it to take a photo containing the measurement on a patient’s monitor with an arterial line; subsequently, the participant operated the app for a few minutes and performed a measurement with a photo obtained by themselves, answering the structured questionnaire afterwards.

**Fig. 1:**
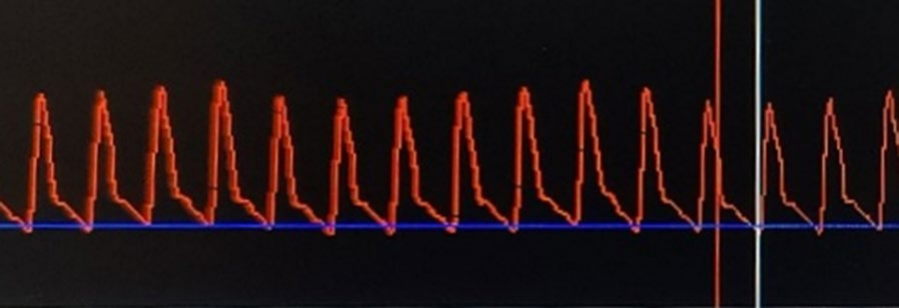
Example of tracing used to measure accuracy—patient with septic shock of pulmonary focus, with hypotension and anuria, receiving vasoactive drugs, with 13% of pulse pressure variation (PPV) measurement

To assess the accuracy of the app's measurement, we used 3 photographs and compared several consecutive measurements by expert judges, as shown in the example below in Fig. 1.

For composing the gold standard, we evaluated the results of pulse pressure variation with a multiparametric monitor and expert PPV manual annotations. (Philips Medical Systems; DX 2020 monitor) in patients with an arterial line, using invasive mechanical ventilation, with 6 mL per kg of tidal volume, without cardiac arrhythmias. The professionals were approached at their respective workplaces (ICU or surgical center) during their daily shifts. The professionals who performed the accuracy assessment were chosen by convenience, while complying with the prerequisites of knowing the software, working in intensive care, and knowing how to assess pulse pressure variation. Eight physicians were selected for this stage of the study.

The categorical quantitative results were presented as percentages and counts and the numerical ones as measures of central tendency. Kolmogorov–Smirnov normality tests were performed for numerical variables. After the abovementioned calculation (in the SUS section) to obtain the final SUS score, the mean score for all participants was calculated. Also, we presented the absolute and relative frequencies of each category in each questionnaire item. The chi-square test was used to verify association for categorical variables. The analysis of variance (ANOVA) method for measuring repeatability and reproducibility was used, which is a measurement system analysis technique that uses a random effect model of ANOVA to evaluate a measurement system. The evaluation of a measuring system is not limited to rulers and scales, for instance, but to all types of measuring instruments, test methods and other measuring systems. The expanded ANOVA R&R measurement system measures the amount of variability induced in measurements by the measurement system itself and compares it with the total variability observed to determine the feasibility of the measurement system. Values ​​of p < 0.05 were considered significant. The data obtained in the collection were tabulated and analyzed using the software IBM SPSS Statistics for Windows, version 23.0, Armonk, NY: IBM Corp. IBM Corp, released in 2015.

All methods were carried out in accordance with relevant guidelines and regulations. All patients who agreed to participate in the study signed the Free and Informed Consent Form and were previously informed about the conditions and objectives of the study. All of them were free to withdraw from the study at any time, without entailing any kind of damage to their physical or emotional integrity. The National Research Ethics Commission of Brazil (CONEP), in accordance with the attributions defined in CNS Resolution number 466 of 2012 and CNS Operational Standard number 001 of 2013, approved the research project.

## Results

**Fig. 2:**
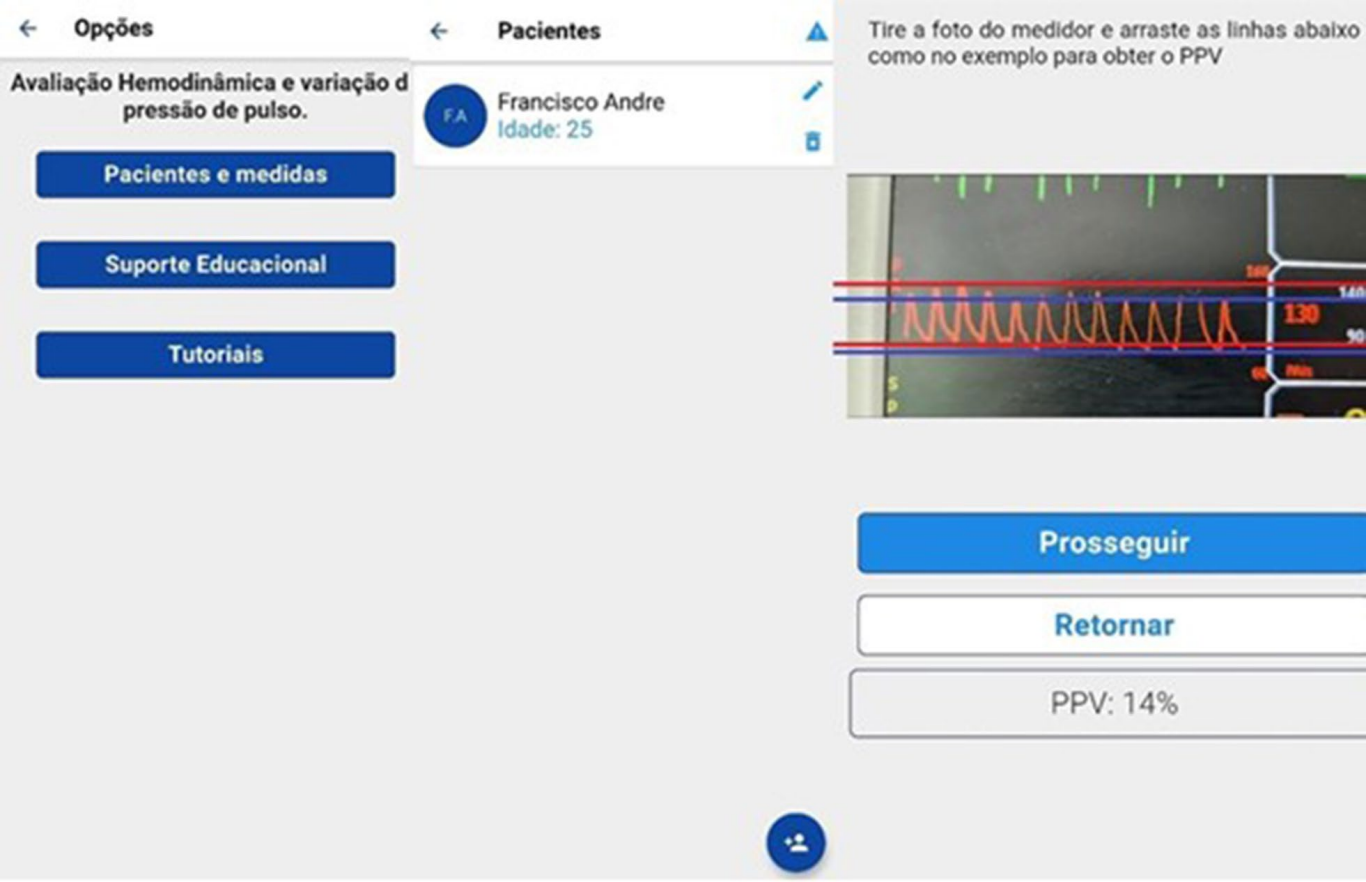
Application home screen and measurement example using the app tool. The red lines should overlap the higher systolic and the higher diastolic pressure (PPMAX) and the blue lines should overlap the lower systolic and the lower diastolic pressure (PPMIN)

The app, temporarily called ‘Hemodynamics’, contains two practical features, an interface to help measure the PPV, an educational support on the hemodynamic monitoring topic, as well as a tutorial explaining the use of the software. The illustration is shown below. By clicking on the item “patients and measurements”, the physician will be redirected to another screen containing the registered patients, where data on the patients and measurements will be stored, with the date and time of the measurement performance. Based on a photo selected from the smartphone library or the cell phone camera, two comparative rulers can be used to measure the PPV; the red colored one should select the pulse wave with the highest amplitude (usually during inspiration) and the blue colored one, the wave with the lowest amplitude (Fig. 2).

**Fig. 3:**
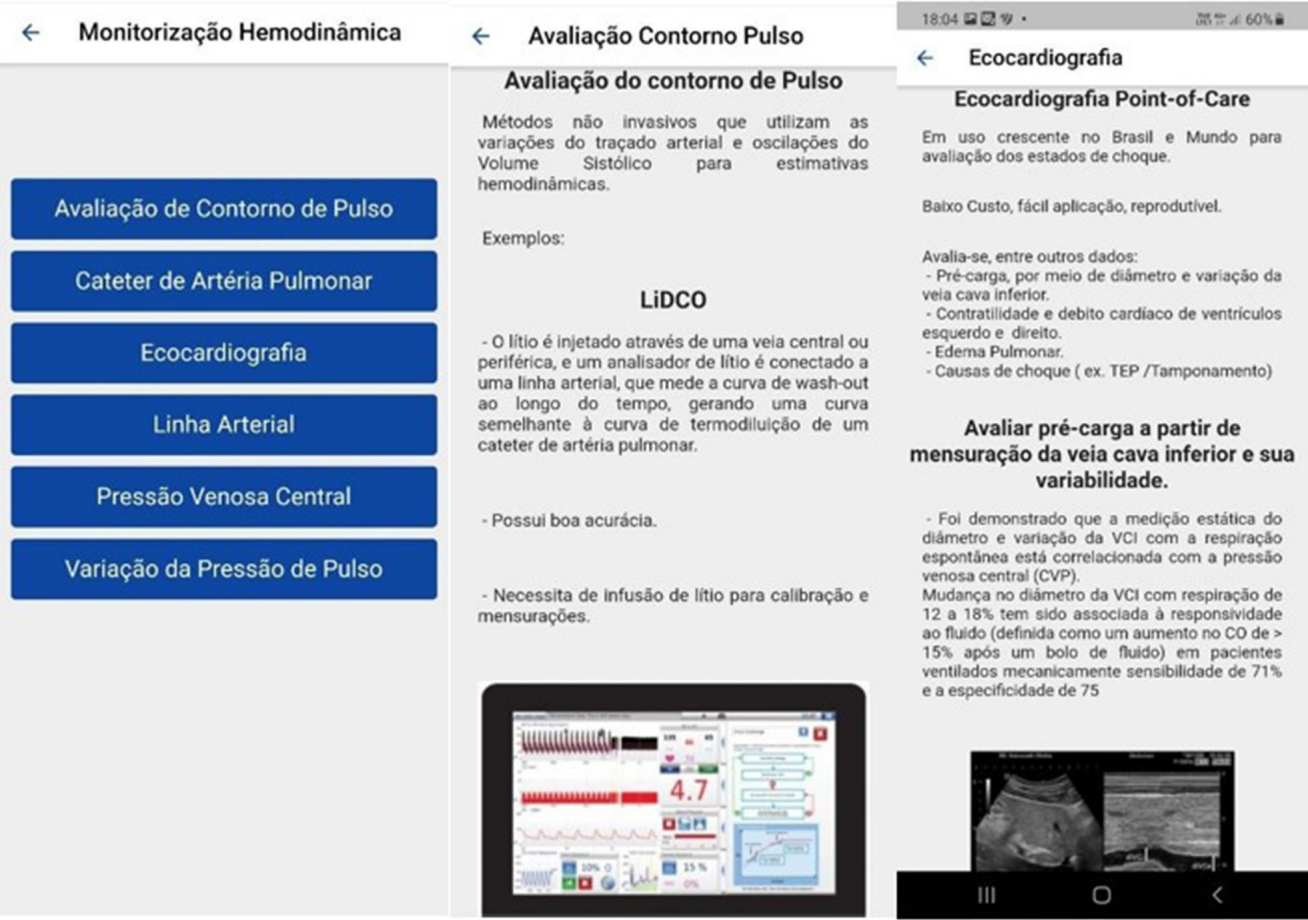
Example of the application educational tool. In the image on the left, list of tool options for hemodynamic monitoring: Pulse contour analysis, pulmonary artery catheter, echocardiography, arterial line, central venous pressure, pulse pressure variation. At the Center, example of the pulse contour evaluation interface. Right image, example of the text and figures from ultrasound use

By clicking on educational support, the app directs the physician to a screen with several examples of hemodynamic monitoring in intensive care for educational purposes and to a session of clinical cases with several emergency and ICU scenarios. The available text is in accordance with the updated guidelines of medical literature, containing information on the topics: Pulse contour assessment (LiDCO®, Vigileo®/FLotrac®, PICCO®), pulmonary artery catheter—with description of the catheter and normal and altered variables, with the differential diagnosis of different types of shock, ultrasonography, central venous pressure and a text on the use of the arterial line and pulse pressure variation (Fig. 3).

The third item directs the physician to two tutorial videos, one on pulse pressure variation and the other on the app utilization. Both videos are available on the internet and can be freely accessed.

We evaluated the developed app regarding its usability according to the SUS in 30 physicians from different specialties, 15 working in Internal Medicine, 4 in General Surgery, 2 in Anesthesiology, 6 in Intensive Care, 2 in Neurology and 1 in Emergency Care. The mean age was 34.6 years (ranging from 25 to 49), with 21 men and 9 women. The mean time since graduation was 8.9 ± 6.7 years, with an average time of experience in the ICU of 5.66 ± 6.58 years, and 29 (96.7%) already routinely use educational apps on cell phones, as shown in Table 1.

**Table 1:** Sample description

Characteristics	N (%) or Median (IQR)
Age (years)	32 (29–43)
Gender	
Male	21 (70)
Female	9 (30)
Time since graduation (years)	6.5 (4–12)
Time of Experience in the Intensive Care Unit	2.5 (1–8)
Have you ever used a medical app for educational purpose?	
No	1 (3.3)
Yes	29 (96.7)

The final SUS score was 86.5, ranging from 60 to 100, with 90 for male and 82.5 for female users, being considered an A+ or better imaginable grade in terms of perceived usability (Table 2).

**Table 2:** Usability assessment according to the SUS scale

Variable	N (%)
I think I would like to use this system frequently	
Agree	14 (46.7)
Strongly agree	16 (53.3)
I found the system unnecessarily complex	
Strongly disagree	17 (56.7)
Disagree	8 (26.7)
Neutral	1 (3.3)
Agree	2 (6.7)
Strongly agree	2 (6.7)
I found the system easy to use	
Strongly disagree	1 (3.3)
Disagree	1 (3.3)
Neutral	1 (3.3)
Agree	7 (23.3)
Strongly agree	20 (66.7)
I think I would need the support of a technical support to be able to use this system	
Strongly disagree	15 (50.0)
Disagree	12 (40.0)
Neutral	–
Agree	2 (6.7)
Strongly agree	1 (3.3)
I found that the various functions in this system were well integrated	
Strongly disagree	–
Disagree	–
Neutral	1 (3.3)
Agree	10 (33.3)
Strongly agree	19 (63.3)
I thought there was a lot of inconsistency in this system	
Strongly disagree	18 (60.0)
Disagree	7 (23.3)
Neutral	1 (3.3)
Agree	3 (10.0)
Strongly agree	1 (3.3)
I imagine that most people would learn to use this system quickly	
Strongly disagree	–
Disagree	1 (3.3)
Neutral	1 (3.3)
Agree	6 (20.0)
Strongly agree	22 (73.3)
I found the system too heavy to use	
Strongly disagree	24 (80.0)
Disagree	5 (16.7)
Neutral	1 (3.3)
Agree	–
Strongly agree	–
I felt very confident in using this system	
Strongly disagree	–
Disagree	1 (3.3)
Neutral	4 (13.3)
Agree	10 (33.3)
Strongly agree	15 (50.0)
I had to learn a lot of things before I could start using this system	
Strongly disagree	12 (40.0)
Disagree	10 (33.3)
Neutral	2 (6.7)
Agree	4 (13.3)
Strongly agree	2 (6.7)

**Fig. 4:**
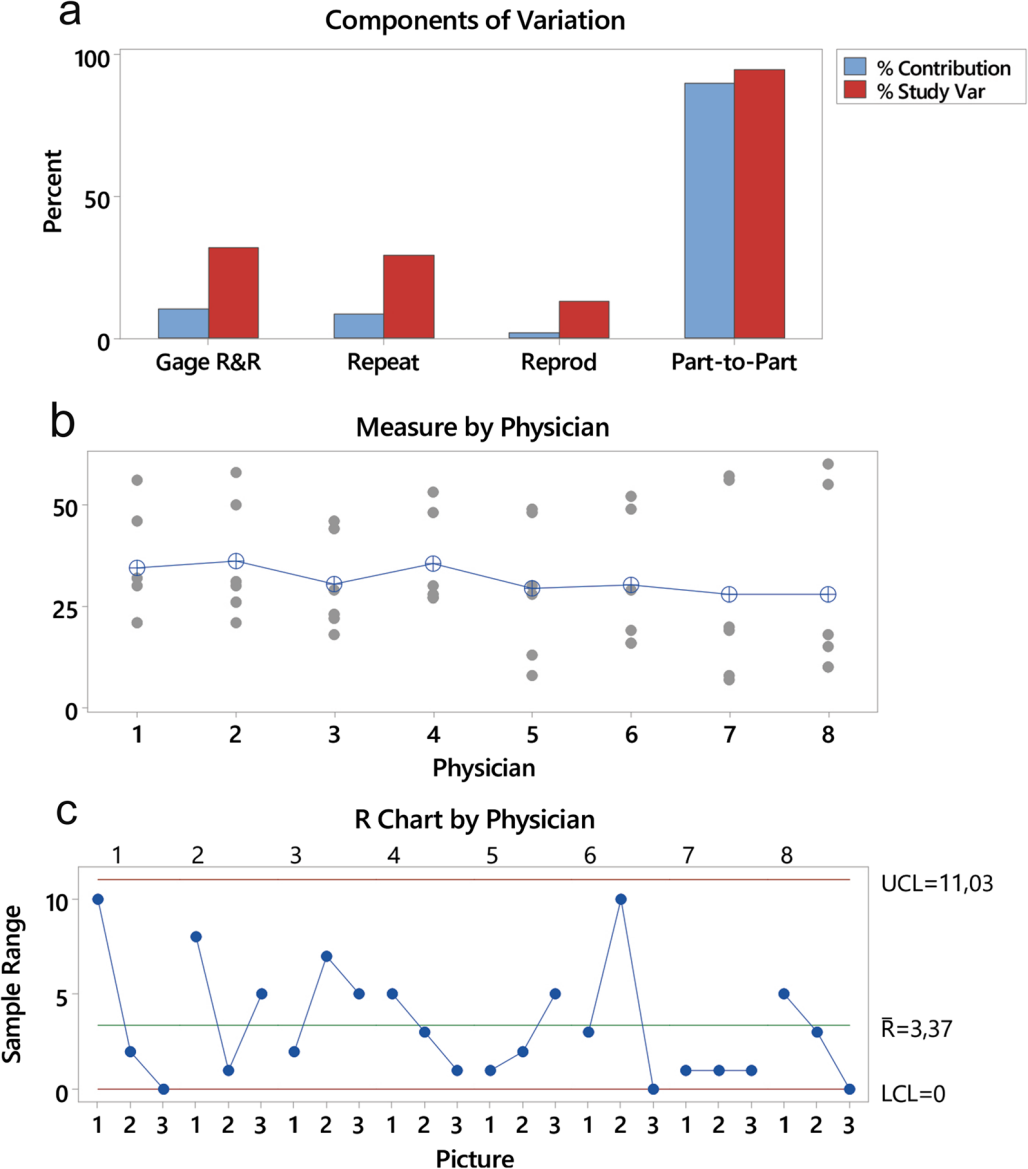
**a** Reproducibility and repeatability analysis. There was roughly 30% variation, due mainly to part-to-part variations. **b** Graph of measurements taken by each participant, showing low variability among examiners. **c** Amplitude graph. Note the random pattern evidencing the absence of biased sampling. *UCL* Upper Control Limit, *LCL* Lower Control Limit, *R* Average Range

In the analysis of reproducibility and repeatability, most of the variation found by the measurement device is due to the adequate variation of the measured objects, with approximately 30% of the variation due to repeatability and reproducibility, with a p-value < 0.001 as shown in Fig. 4a. The graph showing the measurements taken by each participant shows that the average variation of the several measurements taken by the participants was small (Fig. 4b). The sample amplitude graph by participant shows that the mean amplitude, 3.37, was low in relation to the averages of the obtained measurements (Fig. 4c).

When evaluating the free answers of the participants about suggestions and positive and negative aspechts of the app, we observed that the most praised aspects were the practicality of the app, its ease of use, and the quick calculation of the PPV. As criticism and suggestion for improvement, one user commented: “I suggest, when you take a picture of the monitor, a tool that allows you to easily rotate and align the horizontal axis". There was also criticism of the sensitivity of the cell phone touch, which according to some users made it difficult to use the app. No other remarks were mentioned.

## Discussion

In this study, we present the assessment of the qualitative aspects of perceived usability of a mobile app that allows the measurement of pulse pressure variation that had a usability result perceived by the participants of 86%, considered excellent, as well as a good good measure of repeatability and reproducibility.

Despite being a hemodynamic variable with low cost of use and high accuracy in some subgroups of patients, it was found that both in the literature and in practice, PPV is underused. One can infer some reasons for this fact. There is a relative technical difficulty in obtaining data on conventional monitors, despite a minority of modern monitors. In the United States of America, only 15.2% of anesthesiologists use PPV as a volume guide in the operating room, although the same study showed that in 95.4% of high-risk surgeries, the patient has an invasive arterial line. In contrast, 72% routinely use the CVP [15], a static parameter known to be less accurate for fluid evaluation. In this same study, in an intensive care setting, to assess responsiveness, North-American anesthesiologists more frequently use blood pressure, urinary output, clinical experience and CVP, rather than PPV.

The cost associated with modern intensive care is high, comprising up to 30% of total hospital costs and 1% of the gross domestic product in the United States. In Australia, the average cost of a critical patient per day is on average US$4,375.00, approximately R$25,000.00 reais at the current exchange rate [16]. The pulmonary artery catheter adds US$14,400 on average to the cost of a patient's hospital stay [17]. The use of minimally-invasive technology with the FloTrac® system has a cost per patient of around R$1,500.00 reais. With the expansion of mobile telephone technology, a new opportunity for the monitoring of potentially cost-effective intensive care interventions can be evaluated in future economic evaluations.

The first automatic calculation algorithms for PPV were part of commercial monitoring systems such as the PICCO system. In 2004, a new algorithm was described, aiming to estimate the PPV index in arterial pressure [18]. The method uses automatic detection algorithms, data smoothing, and rank order filters to continuously estimate PPV. For this new PPV estimation, the algorithm was described in detail to ensure reproducibility, aiming to allow researchers to implement it in their own software packages and to be able to automatically determine PPV from digitally stored ABP (This new algorithm facilitated the dissemination of the method since, considering its open access feature, the companies that manufacture medical equipment are also free to implement it as part of their monitoring systems. Given the recent developments in this area, several medical device-manufacturing companies have continued to develop monitoring systems that include proprietary algorithms to automatically monitor dynamic fluid responsiveness indicators [19].

The Capstesia® program (Galenic App, Vitoria-Gasteiz, Spain), which can be installed on smartphones or tablets, is a mobile application for pulse wave analysis with cardiac output estimation, using a digital extraction technology [20], with a good estimate of stroke volume variation when compared to the traditional SVV measurement methods [21], showing substantial agreement between cardiac output values ​​produced by the Vigileo® monitor and the app in patients undergoing cardiac surgery [22]. We still do not have an impact analysis on mortality, cost and hospital length of stay with this new monitoring app. This app, however, is not available in Brazil and requires a monthly subscription payment, making it difficult to use it in our country.

Our study evaluated the usability perceived by the participants, being one of the first in the world to present results in this sense. The usability perceived by the participants was 86%, which is considered excellent. Other studies have also evaluated the reproducibility of other applications for measuring PPV, reaching results of 9%, close to those found in this study [20].

The calculation of pulse pressure variation in our app does not use automated algorithms, but a manual measurement. This potentially decreases the sensitivity of the method through the potentiation of errors made by the user, being an important disadvantage of the method. On the other hand, it makes the app lighter and more flexible, not requiring a continuous internet connection, allowing its use in more remote areas.

Considering the social changes regarding the use of smart devices, mobile devices will increasingly become a key component of medical students’ learning and the use of a mobile device as an educational tool has shown to have a positive effect on the acquisition of knowledge and skills among medical students and residents [23].

The lack of apps of similar function does not allow a direct comparison in terms of usability with other articles. Other apps used in smartphones for various functions (cardiac arrest assistance, diabetes management, major burns, among others) use the SUS scale in a similar way for their evaluation [24].

Our study has several limitations. Firstly, the population was studied in two intensive care centers from tertiary hospitals, making a broader generalization of results difficult. Secondly, our evaluation focused mainly on qualitative aspects of perceived usability, and the assessment of the effectiveness of the interventions based on the data provided by the app should be the subject of future investigations. Finally, showing the participants how to use the application immediately prior to the testing session may have introduced bias on the generality of the results. However, the app has a self-instructing session that may fill this gap.

## Conclusions

Our software showed acceptable qualitative aspects of perceived usabilityusability, good repeatability and reproducibilityand potential benefit in teaching and in medical care for critically-ill patients. Comparative studies to analyze its effectiveness in actual clinical practice scenarios are necessary for its wider use.

## Data Availability

The datasets generated and/or analysed during the current study are not publicly available due to Brazilian ethical laws but are available from the corresponding author on reasonable request.
